# Chondroblastoma of the patella with secondary aneurysmal bone cyst, an easily misdiagnosed bone tumor:a case report with literature review

**DOI:** 10.1186/s12891-021-04262-0

**Published:** 2021-04-23

**Authors:** Jianping Zheng, Ningkui Niu, Jiandang Shi, Xu Zhang, Xi Zhu, Jiali Wang, Changhao Liu

**Affiliations:** 1 General Hospital of Ningxia Medical University, 804 Shengli Street, Xingqing District, Yinchuan, 750004 People’s Republic of China; 2 Ningxia Medical University, 1160 Shengli Street, Xingqing District, Yinchuan, 750004 People’s Republic of China

**Keywords:** Chondroblastoma, Patella, Aneurysmal bone cyst, Misdiagnosed, Case report

## Abstract

**Background:**

Chondroblastoma (CB) is a rare, primary, benign bone tumor that commonly affects men aged 15–20 years. It is usually detected in the epiphysis of the long bones, such as the proximal femur, humerus, and tibia. The patella is an infrequent site. CB with secondary aneurysmal bone cyst (ABC) is extremely rare in the patella, which can be easily confused with other common bone tumors of the patella. Thus, it is necessary to make the right diagnosis to get a good outcome.

**Case presentation:**

We have presented here the case of a 30-year-old man who was suffering from anterior knee pain for the past 6 months that had aggravated 2 weeks before the presentation. Osteolytic bone destruction in the patella could be detected in both his X-ray and computed tomography (CT) examinations, while the magnetic resonance imaging (MRI) detected a fluid level. Accordingly, secondary ABC was presumed. We diagnosed the condition as giant cell tumor (GCT) with secondary ABC and, accordingly, performed curettage inside the focus region with autogenous bone grafting following the patient’s medical history, physical manifestations, results of physical and ancillary examinations, and the disease characteristics. However, the intraoperative and postoperative outcomes indicated that the patient’s histopathology was consistent with that of typical CB, suggesting a definitive error in diagnosis. Accordingly, the patient was finally diagnosed with patella CB along with secondary ABC.

**Conclusions:**

Past studies have demonstrated that the 3 commonest bone tumors affecting the patella are GCT, CB, and ABC. CB with secondary ABC can be easily misdiagnosed as GCT with secondary ABC or ABC. Performing incision biopsy or excision biopsy and conducting histological examination may be the most effective method for suspected CB with secondary ABC.

## Background

Chondroblastoma (CB) is a rare, primary, benign bone tumor that originates from the cartilages, accounting for only 1–2% of all primary bone tumor cases reported for the whole body [1]. CB arises from the secondary ossification center of the epiphysis and the epiphyseal plate, and it is common in older children and adolescents, but rare in adults. The ratio of CB occurrence between men and women is approximately 3:2 and its local recurrence rate is approximately 15% [2]. It often affects the proximal epiphysis of the femur, humerus, and tibia [3, 4], but rarely affects the patella [5, 6]. Only a few cases of CB combined with secondary ABC [7] have been reported to date, and they are easily misdiagnosed as giant cell tumors (GCT) or ABC. CB combined with secondary ABC has been reported previously, with the main focus being the treatment regime, albeit not concerning the differential diagnosis with GCT or ABC. The present article reports a patient who was diagnosed as patella CB with secondary ABC and admitted to our hospital for the same; we have presented here a review of the patient’s diagnosis and the potential differential diagnosis.

## Case presentation

A 30-year-old man presented with the complaint of pain in his left knee for the past 6 months that had further aggravated in the past 2 weeks. There was no obvious inducement of the knee pain 6 months ago, and, although it was aggravated after certain activities, the pain remained tolerable. The patient did not seek any treatment because the pain did not affect the patient’s daily life activities. However, when the patient experienced increased pain for the past 2 weeks, he sought medical help from the hospital.

His physical examination revealed obvious tenderness in the left anterior patella, no swelling in the left knee, and no effusion in the joint cavity. Normal color and temperature of the local skin, a negative result on the floating patella test, and no knee instability and locking were observed. He showed normal knee movement. No abnormality in the laboratory test results was recorded before surgery.

**Fig. 1:**
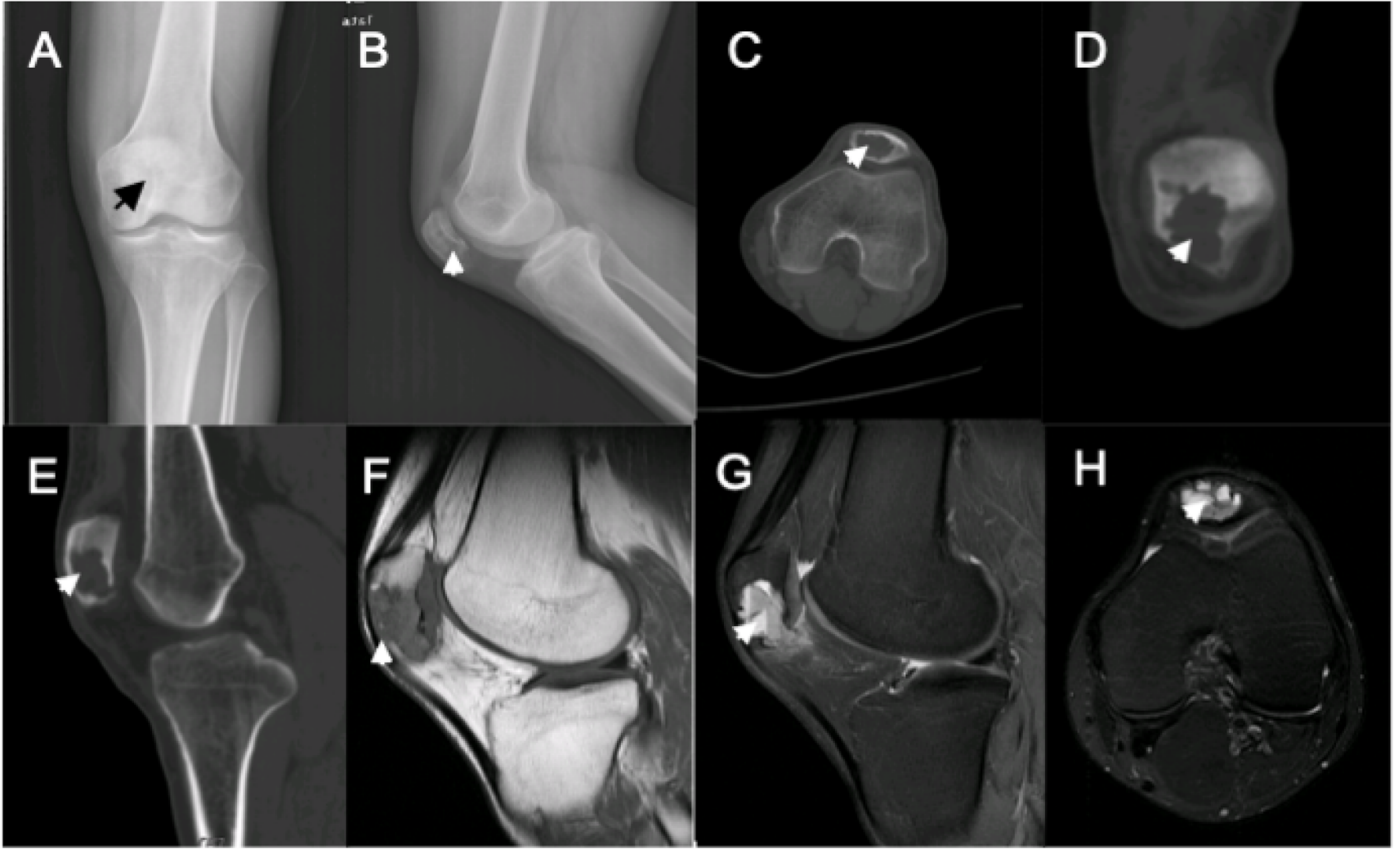
Preoperative imaging results (**a**, **b**). The radioparent lesion in the patella can be seen in the anteroposterior and lateral X-rays (**c**, **d**). The focus with a sharp edge, in a lobulated or worm-eaten shape, was observed in the axial and coronal views of preoperative CT. No boundary or internal calcification was noted in the focus (**e**). At the sagittal view of preoperative CT, the focus was connected with the joint cavity at the junction between the inferior patella and the articular surface (**f-h**). In preoperative MRI, f was T1WI, the patella focus was hypointensity, and g and h were T2WI. The focus was hyperintense and contained the fluid level. Therefore, secondary ABC was suggestive

Imaging examination included anteroposterior and lateral left knee radiographs before surgery. The density of the left patella was found to be decreased uniformly, and the joint space was normal. Preoperative CT revealed osteolytic cystic bone destruction at the middle and lower portions of the left patella, in a lobulated or worm-eaten shape with sharp edges. The local bone cortex had thinned. The focus boundary was clear, and there was no sign of sclerosis at its edge. Preoperative MRI revealed abnormal MRI signals of patchy shadow with a clear margin in the left patella. The T1-weighted images (T1WI) showed hypo-intensity, while the T2-weighted images (T2WI) showed hyperintensity. The fluid level could be observed on the sagittal or axial view of the T2WI, suggestive of ABC as a concern. Preoperative lung CT revealed no neoplasm metastasis focus (Fig. 1).

The condition could be diagnosed as GCT with secondary ABC (Enneking stage II) based on the patient’s age and imaging findings. The surgery was performed after completing the preoperative preparations. However, no needle biopsy was performed before the surgery, for the following reasons:

1. The patient’s age and imaging findings were consistent with those of GCT, which made the preoperative diagnosis credible and eliminated the necessity of preoperative needle biopsy.
2. Only a few samples could be collected by fine-needle aspiration or core needle biopsy due to the small focus or insufficient experience of the operator.
3. The ABC with the small focus identified in the preoperative MRI raised difficulty in collecting sufficient samples.

**Fig. 2:**
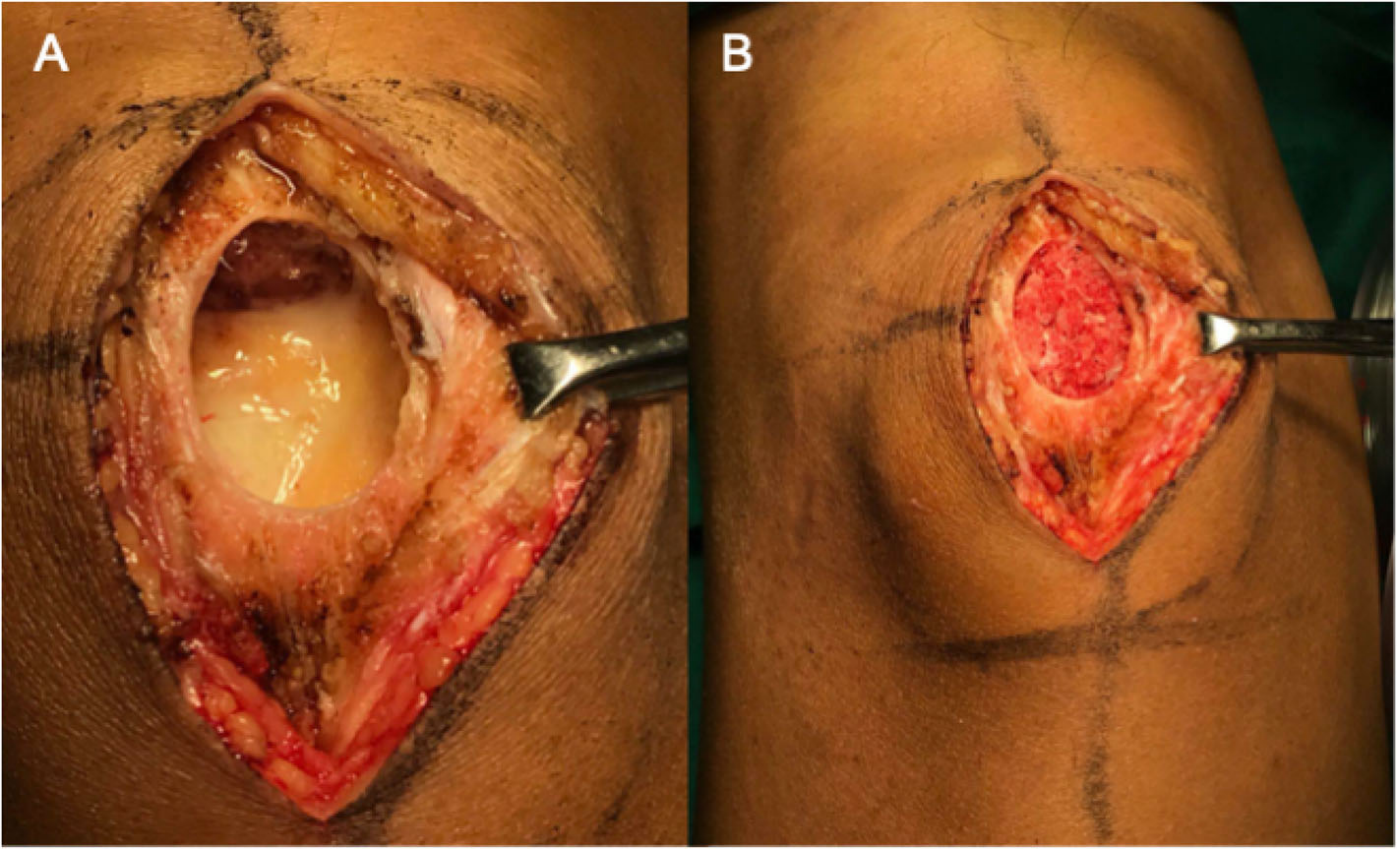
Figures before and after bone grafting at the focus during the surgery (**a**). The focus was debrided through the oval cortical window on the anterior patella. Multiple partitions of the focus were removed and the cartilage surface of the patella was complete (**b**). Autologous bone was grafted at the focus and filled the latter well

The patient was made to lie in a supine position, and his left lower limb and right ilium were disinfected and draped. After approaching the lesion, a cortical window was created. Fresh blood was observed to be oozing out of the focus after the fenestration, and the focus had a multi-dividing shape containing some myxoid substance. The cartilage surface of the patella was relatively complete. A biopsy was obtained for the frozen section to confirm the provisional diagnosis. Through the cortical window, the intralesional curettage of the lesion was extended. Then, a high-speed burr was used to enlarge the tumor cavity, allowing the removal of an additional 1–5 mm of the cavity lining. After repeated irrigation of the tumor cavity with povidone-iodine solution and isotonic saline, autologous iliac bone was harvested from the right ilium, followed by grafting in the focus (Fig. 2) and suturing of the incisions in turn.

**Fig. 3:**
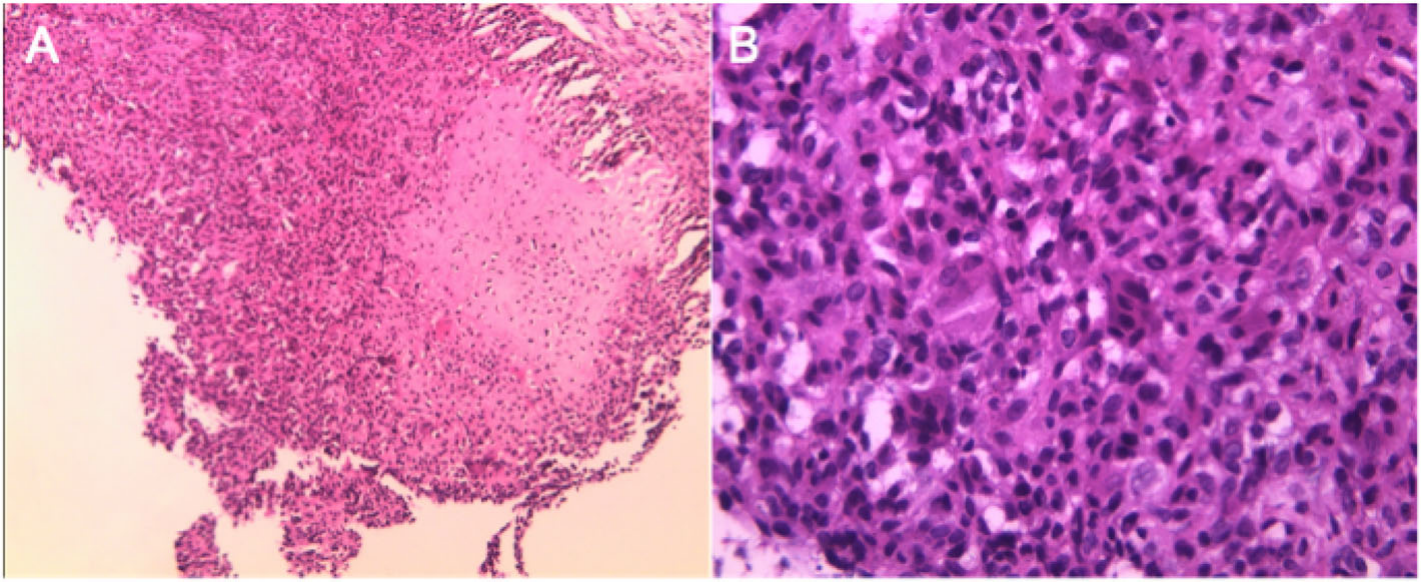
Pathological outcomes of the surgery (**a**). The tumor was composed of confluent proliferating cells in a round and oval shapes, and osteoclast-like multi-nucleated giant cells were scattered throughout the tumor. × 100 (**b**). Under a high-power microscope, the tumor cells showed up as round cells with a clear boundary, while the cytoplasm was light red in color or transparent with nuclear grooves. A pink cartilage matrix was observed in the tumor cells. × 400

The reports for the intra-operative frozen section and postoperative pathological results revealed that the tumor tissues in the left patella were composed of osteoclast-like multi-nucleated giant cells and chondroid matrix. Accordingly, CB was considered (Fig. 3).

**Fig. 4:**
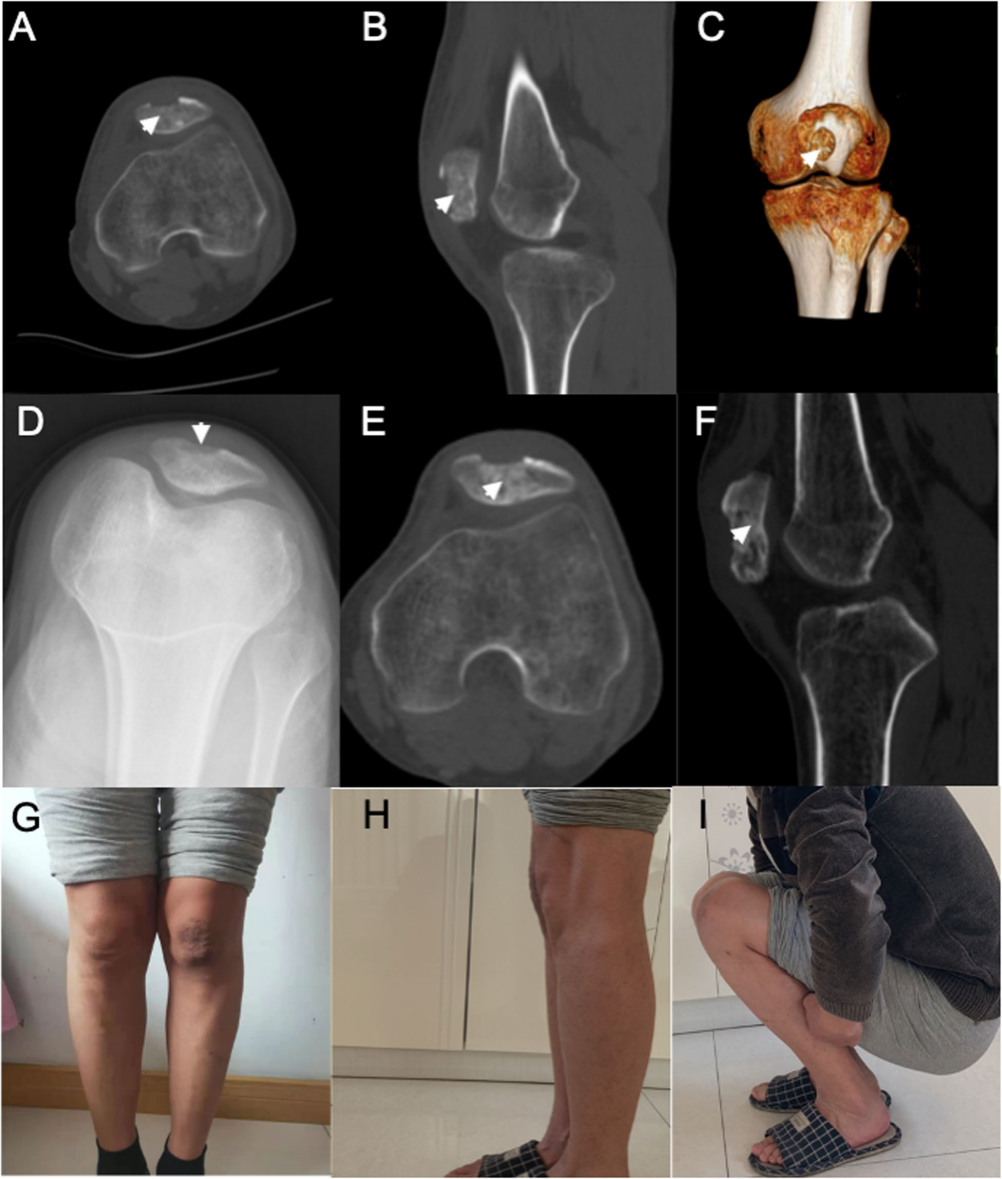
Postoperative imaging data (**a-c**). The fenestration for the focus was found to be reasonable in the postoperative reconstructed, axial, and sagittal CT, and patella-reconstructed CT (**d-f**). X-rays and CT scans showed that the grafted bone had been partly incorporated with the host bone 2 months after the operation (**g-i**). Two months after the operation, the range of motion of the knee joint was normal

The left knee joint was reviewed after the surgery, and the grafted bone at the focus was found to have a good effect on the anteroposterior and lateral films and 3D-reconstructed CT. The static quadriceps exercise was started immediately after the surgery. The knee joint was next fixed with braces at the extension position for 4 weeks, after which the functional exercise for the knee was started. Two weeks after the operation, the pain was completely relieved. The range of motion of the knee joint was normal 2 months postoperatively. The imaging showed that the grafted bone had been partly incorporated with the host bone (Fig. 4). The patient is satisfied with the current outcomes.

## Discussion and conclusions

CB is a benign bone tumor that commonly affects the epiphysis of the long bones. Kolodny et al. reported CB in 1927 and described it as GCT-containing cartilage [8]. This disease was also depicted as a cartilage-like GCT [9] in the epiphysis or as calcified GCT [10] before it was officially named CB in the year 1942 [11]. Hence, there exists an extensive overlap between them in some aspects. Laitinen et al. claimed that [12] CB, GCT, and ABC share similar clinical presentations and imaging characteristics. Besides, secondary ABC is further observed in CB and GCT [13, 14], which adds to the difficulty of accurately diagnosing such a complex disease. Therefore, it is critical to accurately diagnose this disease to design an optimized treatment plan. So far, reports on patellar CB have mainly focused on its treatment, and there is no report on its differential diagnosis. This is the first report on how to correctly diagnose CB with secondary ABC from the perspective of misdiagnosis. Our study suggests that it is extremely easy to be misdiagnosed as GCT or ABC when patella CB is combined with secondary ABC. An incision biopsy or excision biopsy and histological examination can help confirm the diagnosis and guide treatment for the suspected CB with secondary ABC.

Accurate diagnosis is a prerequisite for a good prognosis. According to the literature, three bone tumors commonly affect the patella, namely, GCT, CB, and ABC [12, 14]. Among these, GCT is the commonest, accounting for 33% of all patella bone tumor cases, followed by CB and ABC at 16 and 5%, respectively. Similarities and differences between them in terms of onset characteristics, clinical presentations, physical examinations, imaging findings, histone H3.3 mutation, pathology, and treatments are summarized in Table 1 [5–7, 15–26]. Although there are many similarities, each has its unique imaging and pathological characteristics. CB was featured as an integrated sclerotic bone at the margin of focus and scattered calcified lesion, wherein the focus could extend to the subchondral bone, although it seldom entered the joint space [20]. Soap-bubble-like changes could be seen in some GCT lesions, which led to the destruction of the bone. As GCT is invasive to a certain extent, it is often complicated by certain pathological fractures. Occasionally, it even breaks through the cortical bone and invades the nearby tissues [21]. The appearance of multi-chamber lesions indicates secondary ABC [5, 22]. Based on the above-mentioned unique imaging findings and different onset characteristics of the disease, a preliminary correct diagnosis can be made. However, when CB or GCT is combined with secondary ABC, its imaging characteristics will become atypical, mainly manifested as osteolytic lesions and liquid levels. Therefore, it is easy to be misdiagnosed. The case we reported just illustrates this point. From the age of onset (30 years old), the incidence of patella bone tumors (common in GCT), and imaging findings (osteolytic lesions with fluid level), the case should first be diagnosed as GCT with secondary ABC. But we made the wrong diagnosis before the pathological results came out. The reason is that we have paid too much attention to the characteristics of common tumors that occur in the patella while ignoring the incidence of rare tumors in the patella and the imaging atypicality caused by secondary ABC. Therefore, for such diseases, we need to rely on pathological or genetic results to further confirm the diagnosis.

**Table 1:** Differences and similarities between GCT, CB, and ABC of the patella

	GCT	CB	ABC
Incidence rate	33%	16%	5%
Age (years)	20–40	15–20	10–20
Common location	The distal femur, proximal tibia, and distal radius	Proximal epiphysis of the femur, humerus, and tibia	Epiphysis of the femur and tibia
Clinical presentation	Knee pain	Knee pain	Knee pain
Physical examination	Tenderness	Tenderness	Tenderness
Imaging findings	X: osteolytic lesion, soap bubble appearance, non-sclerotic margin, and radiolucent lesion; CT: osteolytic lesion; MRI: osteolytic lesion, hypointensity on T1WI, and hyperintensity on T2WI	X: osteolytic lesion with well-defined sclerotic margin, lobulated rims, and thinned cortex; CT: osteolytic lesion with septation, sclerotic margins, and some intralesional calcifications; MRI: lobulated lesion, iso/hypointensity on T1WI, and mixed intensity or hyperintensity focus on T2WI	X: geographic osteolysis, smooth borders, thinned cortices, and intact articular surface; CT: fluid-filled multiseptate cavities without intralesional calcifications; MRI: lobulated lesion with a fluid-filled cyst, hypointensity on T1WI, and hyperintensity on T2WI
Histone H3.3 mutation	*H3F3A*	*H3F3B*	–
With secondary ABC	Yes	Yes	–
Pathology	Numerous giant cells, short spindle-shaped cells, bone tissue calcification, and a few mitotic figures	Proliferating chondroblast with chondroid matrix, some multinucleated giant cells, “coffee bean” nucleus	Necrosis and hemorrhagic cystic cavities or red cells
Treatment	Intralesional curettage (benign GCT) or patellectomy with adjuvant treatment (aggressive GCT)	Intralesional curettage followed by bone grafting	Intralesional curettage followed by bone grafting

Although pathological results can assist in making the correct diagnosis, accurate pathological results depend on effective biopsy techniques. Therefore, the selection of the optimized biopsy technique becomes crucial. Currently, the frequently used biopsy technologies mainly include fine-needle aspiration, core needle biopsy, incision biopsy, and excision biopsy. Owing to the involvement of small trauma, fewer complications, lower cost, and ease of operation, the first two technologies are favored by bone oncologists [27, 28]. However, several other factors affect the diagnostic accuracy with the use of these two approaches when combined with secondary ABC. For fine-needle aspiration biopsy, cell samples are mainly collected. Although it can be taken from multiple sites to increase the accuracy of diagnosis, the accuracy rate is approximately 70%. Such a low accuracy rate may be related to the atypical cytological characteristics of ABC, which eliminates the need for fine-needle aspiration biopsy for preoperative diagnosis of patients with ABC. A core needle biopsy can collect more samples, including cells and tissues, while this technique can only increase the diagnostic accuracy rate to 81%, and it is related to the size of the lesion. When the focus size is ≥3 cm, the diagnosis accuracy rate can reach up to 89.2%, but when focus size is < 3 cm, it can only reach up to 73.4% [29, 30]. Incision biopsy has been regarded as the gold standard for diagnosis because of its good sample-collection system and high positive diagnosis rate. Considering secondary ABC with a small focus of this patient before the surgery, incision biopsy was adopted instead of preoperative needle biopsy. The postoperative pathological results were highly consistent with those of the specific manifestations [5–7, 19] (proliferated chondroblasts in the laminar regions accompanied by cartilage matrix and some multi-nucleated cells. Calcification may also appear in some areas) of CB. In the treatment process of this case, the accurate pathological diagnosis through incision biopsy not only revised the initial treatment plan in time but also reduced the adverse effects caused by the low accuracy of percutaneous biopsy.

In case of insufficient tissues available for biopsy or case of the lack of chondrogenic differentiation area in the limited samples, monitoring the K36M mutation on *H3F3A* and *H3F3B* by immunohistochemistry or polymerase chain reactions is an effective technique. Past studies have shown that [25, 26] histone H3.3 mutations exist in both GCT and CB, especially *H3F3A* mutations in GCT and *H3F3B* mutations in CB. This feature has encouraged the development of new diagnostic tools [31, 32] that employ gene sequencing for *H3F3A* and *H3F3B* or uses the specific antibodies of G34W and K36M mutation for immunohistochemical analysis to enable distinguishing between CB and GCT. However, the wide application of these tools in clinical practice can only be realized after a long time.

Although the case was misdiagnosed before the operation, the diagnosis was accurately corrected due to the reasonable choice of biopsy technique for pathological diagnosis. In the end, the patient received reasonable treatment and obtained a good prognosis.

In conclusion, CB of the patella can be distinguished from GCT or ABC in terms of disease characteristics and imaging findings. However, when CB is combined with secondary ABC, it is easily misdiagnosed because its typical imaging features will overlap with the imaging findings of other tumors that occur at this site, and then become atypical or even be masked. By reviewing recent studies and referring to the results of this case report, we conclude that an incision biopsy or excision biopsy and histological examination may be the most effective diagnostic method for suspected CB with secondary ABC.

## Data Availability

The datasets used and/or analyzed during the current study are available from the corresponding author on reasonable request.

## Abbreviations

CB: Chondroblastoma
ABC: Aneurysmal bone cyst
CT: Computed tomography
MRI: Magnetic resonance imaging
GCT: Giant cell tumor
T1WI: T1 weighted images
T2WI: T2 weighted images
